# Supplementary material for: A new role for PHYHD1 and related dioxygenases: demethylation of 2′-O-methylated nucleosides
Source: Nucleic Acids Res. 2025 Dec 17;53(22):gkaf1379. doi: 10.1093/nar/gkaf1379 (PMC12709175; doi:10.1093/nar/gkaf1379)
Supplement: gkaf1379_Supplemental_Files [file gkaf1379_supplemental_files.zip › FJS-SUPPLEMENTARY-DATA.pdf]

A new role for PHYHD1 and related dioxygenases: demethylation of 2′-*O*-methylated nucleosides

Justas Stonkus[1,*], Rasa Rutkienė[1], Rita Meškienė[1], Martyna Jasiūnienė[1], Agota Aučynaitė[1], Laura Kalinienė[1], Justas Lazutka[2], Darius Balčiūnas[2,3], Giedrius Vilkaitis[2], and Rolandas Meškys[1,*]

[1] Institute of Biochemistry, Life Sciences Center, Vilnius University, Vilnius LT-10257, Lithuania

[2] Institute of Biotechnology, Life Sciences Center, Vilnius University, Vilnius LT-10257, Lithuania

[3] Department of Biology, Temple University, Philadelphia, PA 19122, USA

* To whom correspondence should be addressed. Tel: +370 6 227 0979; Email: justas.stonkus@gmc.stud.vu.lt.

SUPPLEMENTARY DATA

Molecular cloning and plasmid construction

Construction of pLATE31-fjs

The gene coding for FJS was amplified from pUC19-fjs plasmid, isolated from a soil-derived metagenomic library, using Phusion DNA polymerase (Thermo Fisher Scientific, Lithuania, #F632L) with pLATE31-fjs-Fw and pLATE31-fjs-Rv primers. The primers were designed for cloning using aLICator LIC Cloning and Expression Kit 3 (Thermo Fisher Scientific, Lithuania, #K1291). The amplified gene was cloned into the pLATE31 vector, generating the pLATE31-fjs plasmid, containing the gene coding for FJS with a C-terminal His-tag (GHHHHHHG), the sequence of which was verified by Sanger DNA sequencing (Genewiz Azenta, Germany).

Construction of pLATE31-phyhd1-D-rerio and pLATE31-phyhd1X1-D-rerio

Zebrafish (*Danio rerio*) cDNA was synthesised using Maxima H Minus cDNA Synthesis Master Mix (Thermo Fisher Scientific, USA, #M1662) from wild-type AB zebrafish RNA. The amplification of the PHYHD1 gene from zebrafish cDNA was done using Phusion DNA polymerase (Thermo Fisher Scientific, Lithuania, #F632L) with pLATE31-phyhd1-D-rerio-Fw and pLATE31-phyhd1-D-rerio-Rv primers designed for cloning using aLICator LIC Cloning and Expression Kit 3 (Thermo Fisher Scientific, Lithuania, #K1291). DNA sequencing (Genewiz Azenta, Germany) results showed that the cloning generated two different plasmids – one containing the sequence for the canonical *Danio rerio* PHYHD1 (NP_001007446.1) and the other containing the sequence for *Danio rerio* PHYHD1 isoform X1 (XP_009293397.1), both with a C-terminal His-tag (GHHHHHHG).

Construction of pET-21 vectors with genes coding for homologous protein

Genes encoding homologous proteins were cloned into pET-21 expression vectors containing a 5′-ATTTTGTTTAACTTTAAGAAGGAGATATACAT-3′ leader sequence and a C-terminal His-tag (LEHHHHHH). The gene sequences were codon-optimised for expression in *Escherichia coli* and synthesised by Twist Bioscience (USA).

Construction of pET-21-phyhd1C-H-sapiens

The pET-21 vector with the gene coding for PHYHD1 isoform A from *Homo sapiens* was amplified using Phusion DNA polymerase with PHYHD1C-Fw and PHYHD1C-Rv primers. The amplified linear DNA was purified from agarose gel, phosphorylated using T4 polynucleotide kinase (Thermo Fisher Scientific, Lithuania, #EK0032), and circularised using T4 DNA ligase (Thermo Fisher Scientific, Lithuania, #EL0012). The sequence of the resulting plasmid insert was verified by Sanger DNA sequencing (Genewiz Azenta, Germany).

Supplementary Table S1. Sequences of primers used for molecular cloning, plasmid construction, and zebrafish genotyping.

PRIMER	SEQUENCE 5′ – 3′
pLATE31-fjs-Fw	AGAAGGAGATATAACTATGTCTGATTTCGAAGTCACG
pLATE31-fjs-Rv	GTGGTGGTGATGGTGATGGCCAGCTTGCTTTTCGGCCTTC
pLATE31-phyhd1-D-rerio-Fw	AGAAGGAGATATAACTATGGATGTCTTGACAGACCAAG
pLATE31-phyhd1-D-rerio-Rv	GTGGTGGTGATGGTGATGGCCGGTGTAAAGGGAAGGAAACGG
PHYHD1C-Fw	CAACCACACTTTGGCGGCGAAG
PHYHD1C-Rv	TTGCACCTTAAAGCTGTGCGTAATG
phyhd1-D-rerio-genotyping-Fw	AAATGAATATAAATCTGACCTCTGA
phyhd1-D-rerio-genotyping-Rv	CACATAAGCACATGCATGACTCTGA

FJS multimericity analysis

FJS multimericity was analysed using gel-filtration chromatography on a Superdex 200 Increase 10/300 GL column (Cytiva, Sweden, #28990944). Molecular weight standards – including carbonic anhydrase from bovine erythrocytes (BCA), bovine serum albumin (BSA), and apoferritin from equine spleen – were obtained from Gel Filtration Markers Kit for Protein Molecular Weights 29,000-700,000 Da (Sigma-Aldrich, USA, #MWGF1000-1KT).

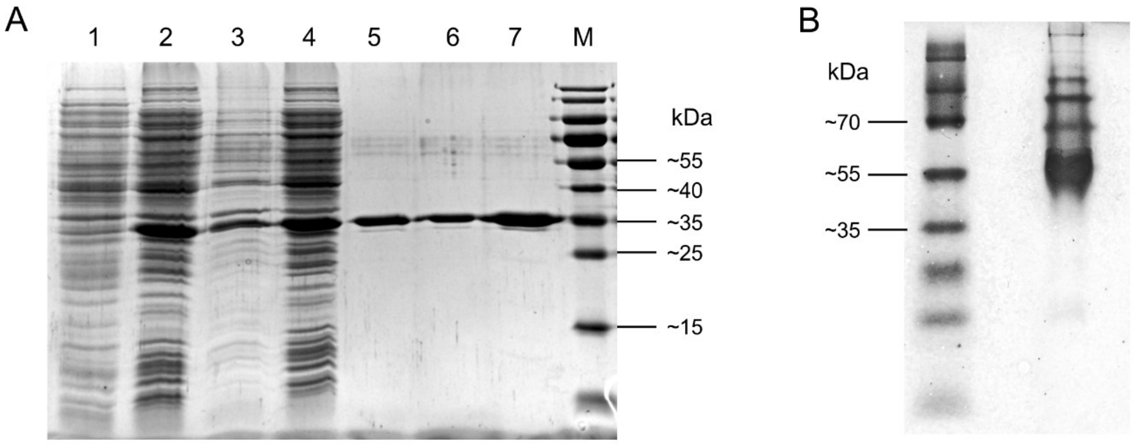

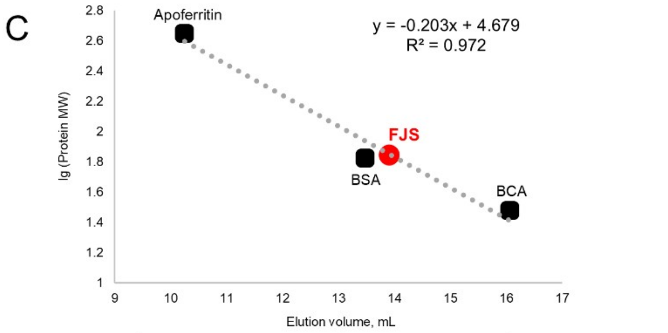

Protein	Elution volume, mL	lg (MW)	MW, kDa
Apoferritin	10.252	2.6464	443
BCA	16.065	1.4771	30
BSA	13.474	1.8224	66.4
FJS	13.910	1.8506	70.9

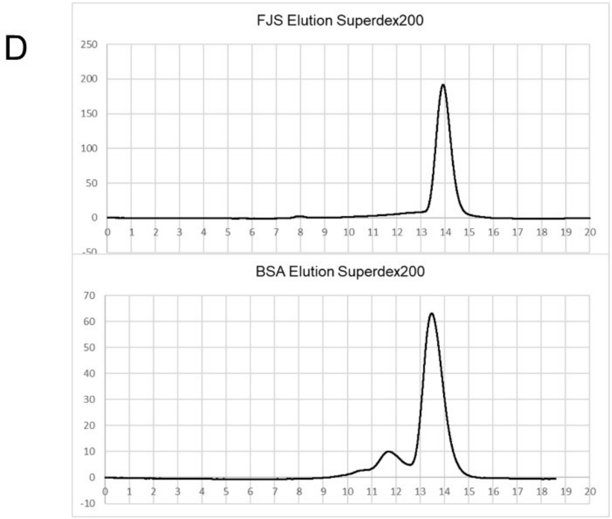

Supplementary Figure S1. (**A**) Initial purification and multimeric state analysis of His-tagged FJS. Recombinant FJS was overexpressed in *E. coli* HMS174(DE3). SDS PAGE was used to analyse the protein at various purification steps: 1 – control lysate of *E. coli* HMS174 (DE3), 2 – lysate of *E. coli* HMS174 (DE3) expressing FJS, 3 – insoluble cell lysate fraction, 4 – soluble cell lysate fraction, 5 – FJS purified on a HiTrap Chelating HP column (Cytiva), 6 – FJS desalted on a HiTrap Desalting column (Cytiva), 7 – FJS concentrated using centrifugal filter units with a 10 kDa MWCO membrane, M – protein molecular weight marker. (**B**) Native (non-denaturating) gel electrophoresis of purified FJS indicated that the recombinant protein (monomer MW 32.9 kDa) predominantly exists as a dimer, migrating between 55 and 70 kDa bands of the molecular weight ladder. (**C**) Gel-filtration chromatography estimated the molecular weight of purified recombinant FJS to be approximately 70.9 kDa, supporting the dimeric state observed in native gel analysis. (**D**) FJS gel filtration chromatogram with BSA gel filtration chromatogram as a reference.

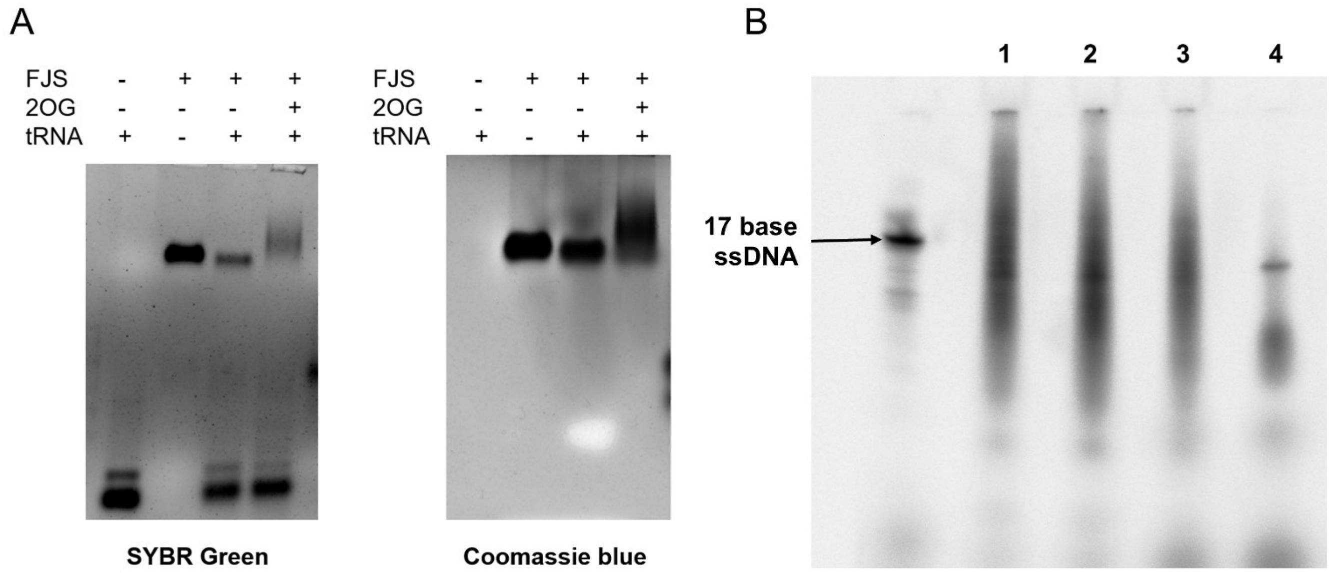

Supplementary Figure S2. (**A**) EMSA experiment indicating that FJS is stained not only by Coomassie blue, but also by SYBR Green dye.

(**B**) Analysis of nucleic acids bound to purified recombinant FJS. Nucleic acids were extracted following protein digestion with Proteinase K. Four extraction and digestion conditions were tested:

1 – extraction with acidic (pH 4.5 – 5.0) ROTI Aqua-Phenol for RNA isolation (Carl Roth, Germany, #A980.2)

2 – extraction with basic (pH 7.5 – 8.0) ROTI Phenol for total nucleic acid isolation (Carl Roth, Germany, #0038.3)

3 – extraction with acidic phenol followed by DNase I digestion (Thermo Fisher Scientific, Lithuania, #EN0521)

4 – extraction with basic phenol followed by RNase A digestion (Thermo Fisher Scientific, Lithuania, #EN0531)

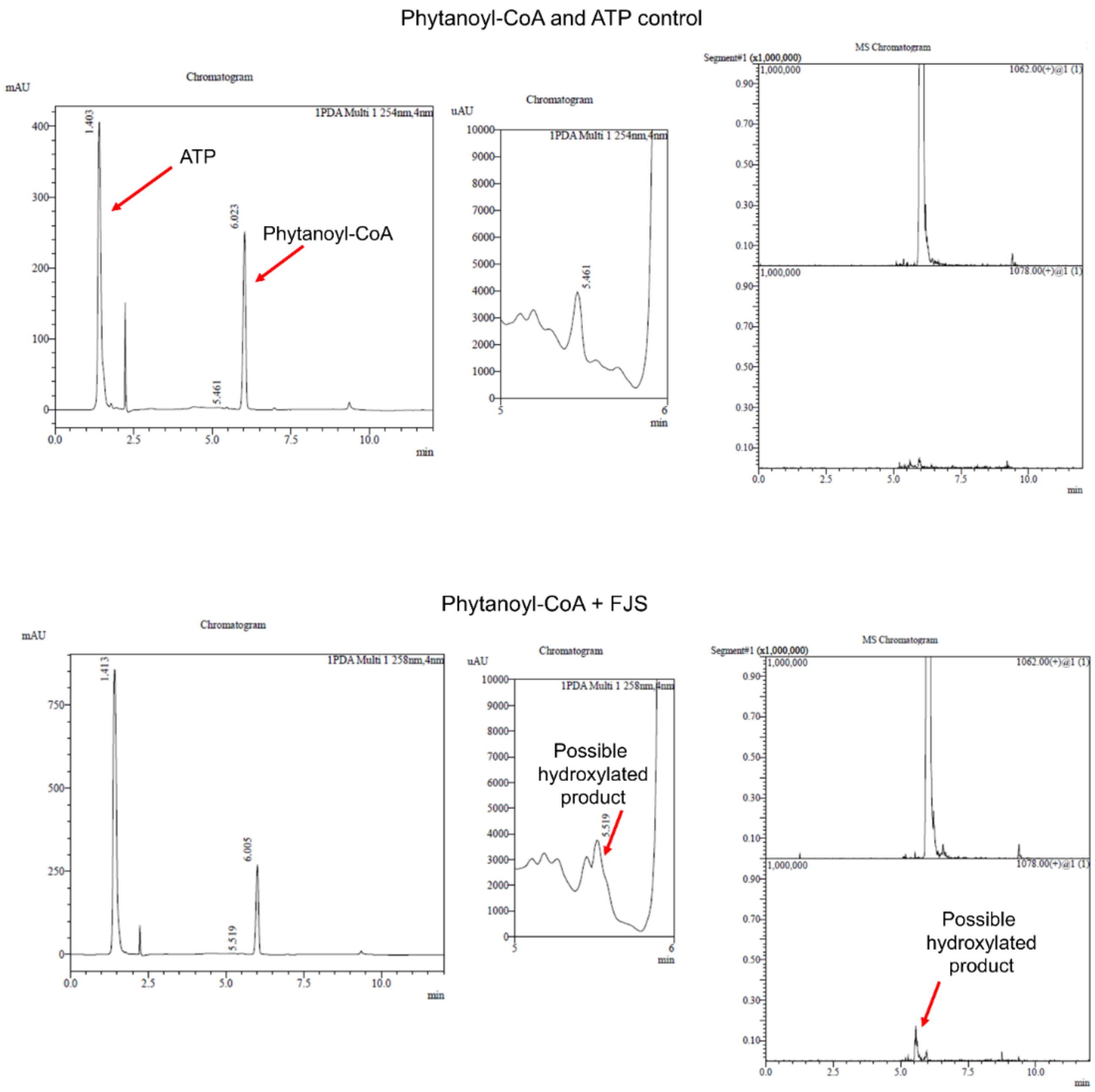

Supplementary Figure S3. HPLC-MS analysis of FJS reaction with phytanoil-CoA. The reaction mixture was based on reactions conducted with human PHYH (1) and consisted of 4 mg/mL FJS, 1 mM FeCl2, 2 mM 2-oxoglutarate, 10 mM ascorbate, 4 mM ATP, 0.05 mM phytanoyl-CoA, 10 µM TCEP, 0.44 mM β-cyclodextrin in 50 mM Tris-HCl buffer, pH 8.0. The reaction mixture was incubated for 12 h at 30°C.

A

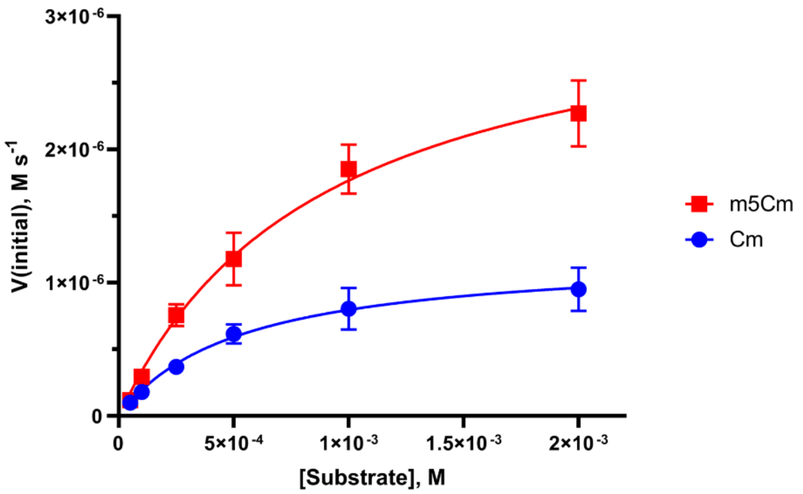

Substrate	kcat, s^{-1}		Km, M		Vmax, M s^{-1}
	Value	SEM	Value	SEM	Value
**m5Cm**	1.10×10^{-1}	8.45×10^{-3}	8.89×10^{-4}	1.36×10^{-4}	3.34×10^{-6}
**Cm**	4.00×10^{-2}	2.89×10^{-3}	5.25×10^{-4}	1.01×10^{-4}	1.22×10^{-6}

B

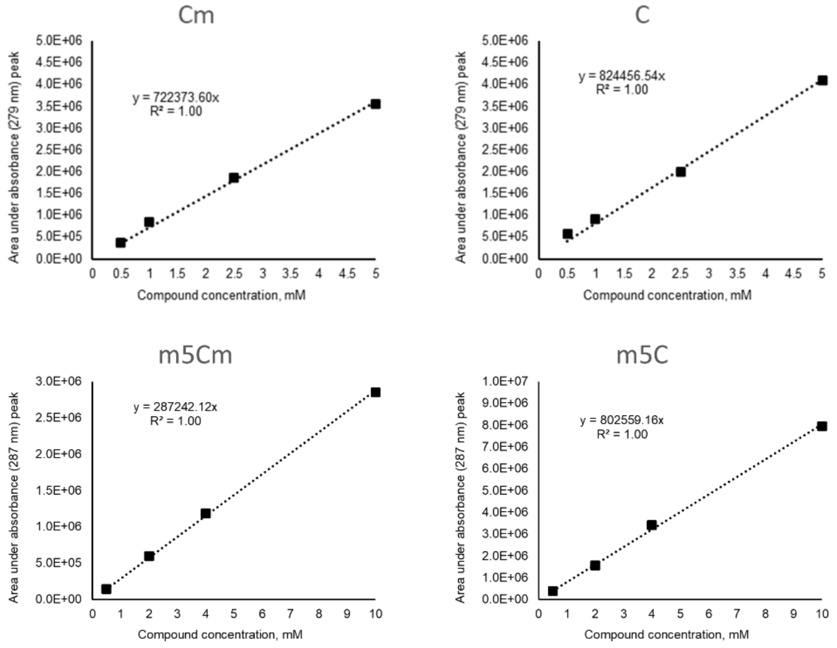

Supplementary Figure S4. (**A**) Kinetic analysis of FJS at 1mg mL^{-1} using Michaelis-Menten kinetics. Reaction mixtures contained purified FJS, the primary substrate, 10 mM 2-oxoglutarate, 0.5 mM $FeSO_4$ and 1 mM ascorbate. Reactions were stopped by the addition of an equal volume of acetonitrile and analysed using HPLC-MS. Initial reaction velocities were determined from at least three points. Substrates tested included 2′-*O*-methyl-5-methylcytidine (m5Cm, n = 19) and 2′-*O*-methylcytidine (Cm, n = 24). (**B**) Standard calibration graphs for 2′-*O*-methylcytidine (Cm), cytidine (C), 2′-*O*-methyl-5-methylcytidine(m5Cm), and 5-methylcytidine (m5C).

A

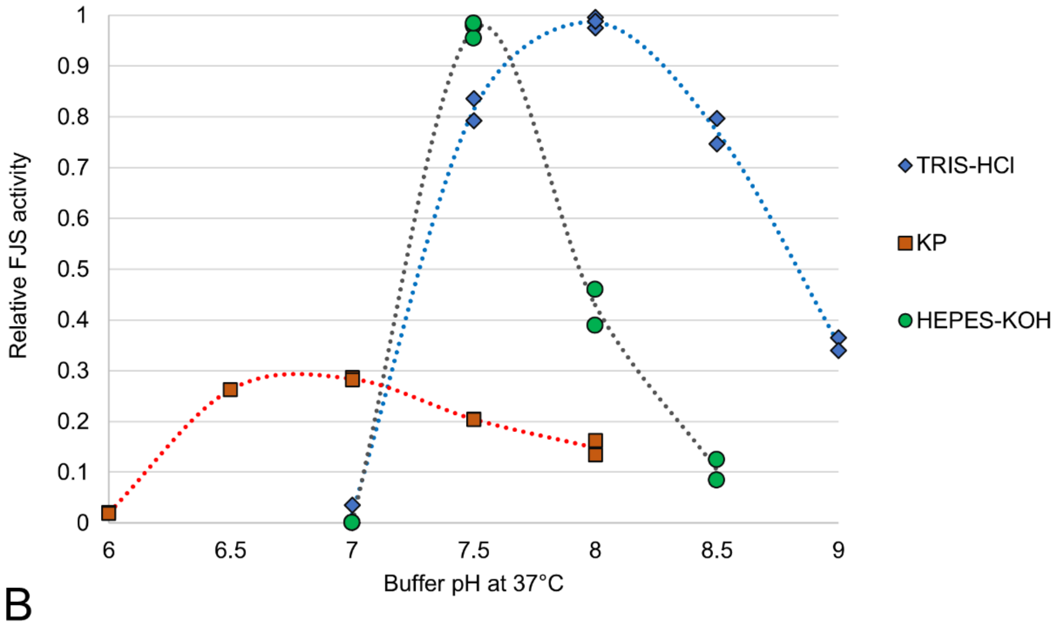

B

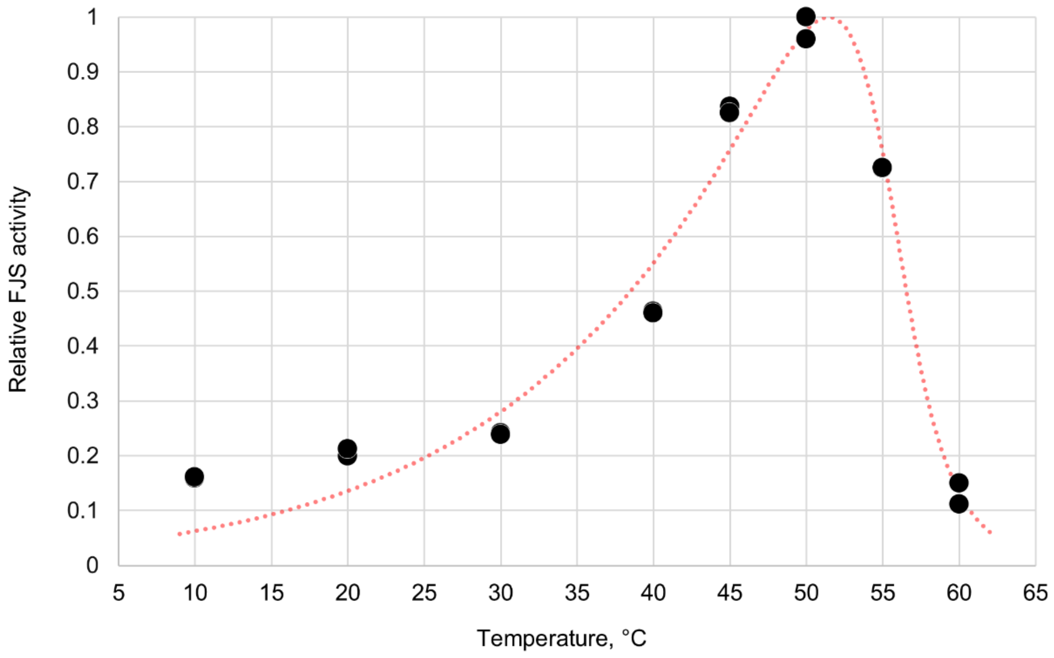

Supplementary Figure S5. Effect of buffer conditions and temperature on FJS catalytic activity. (**A**) The activity of purified recombinant FJS as a function of buffer type and pH. Reactions were conducted at 37°C in 100 mM buffers of varying pH. Note that Tris-HCl buffer at a pH value of 7.5 at 37°C corresponds to a pH value of 8.0 when measured at room temperature. Each 40 µL reaction mixture contained 15 µM purified recombinant FJS, 5 mM 2'-*O*-methylcytidine, 10 mM 2-oxoglutarate, 0.5 mM $FeSO_4$ and 1 mM ascorbate. (**B**) Temperature dependence of FJS catalytic activity. Maximum substrate demethylation (5 mM 2′-*O*-methylcytidine) was observed at 50°C.

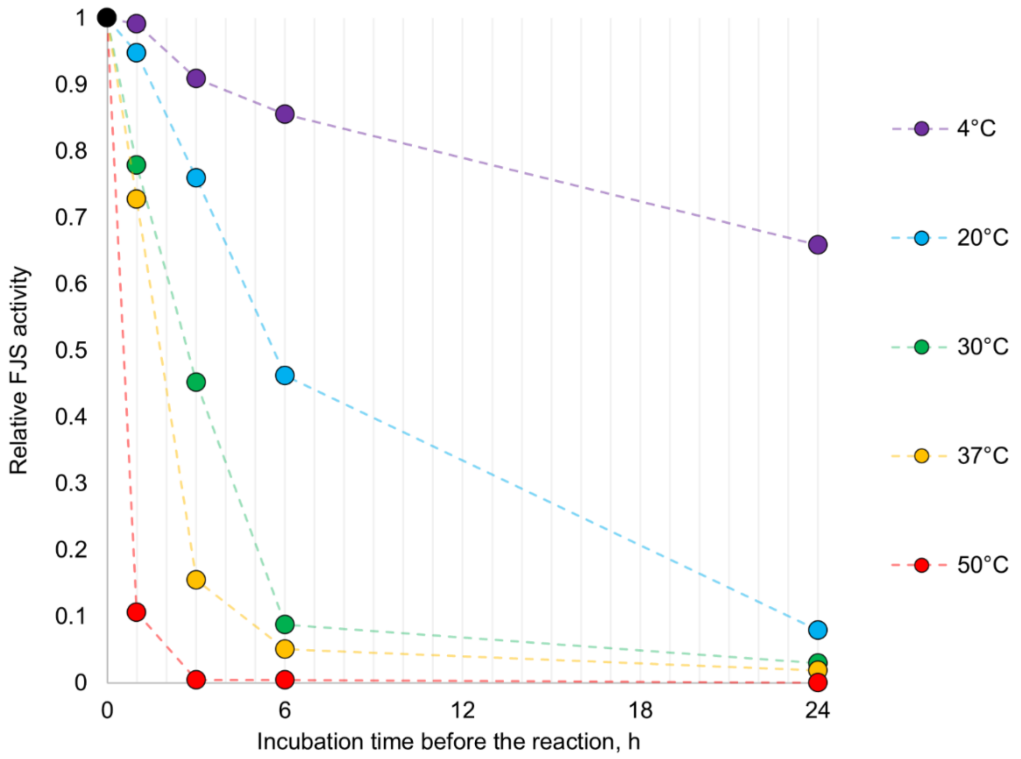

Supplementary Figure S6. Thermal stability analysis of purified recombinant FJS. FJS thermal stability was assessed by measuring relative enzymatic activity, expressed as the percentage of demethylated 2′-*O*-methylcytidine. The enzyme was preincubated in 50 mM Tris-HCl buffer containing 10 % glycerol. Following incubation, reactions were carried out at 50°C for 30 minutes. Each reaction mixture contained 120 µM purified recombinant FJS, 5 mM 2'-O-methylcytidine, 10 mM 2-oxoglutarate, 0.5 mM $FeSO_4$ and 1 mM ascorbate.

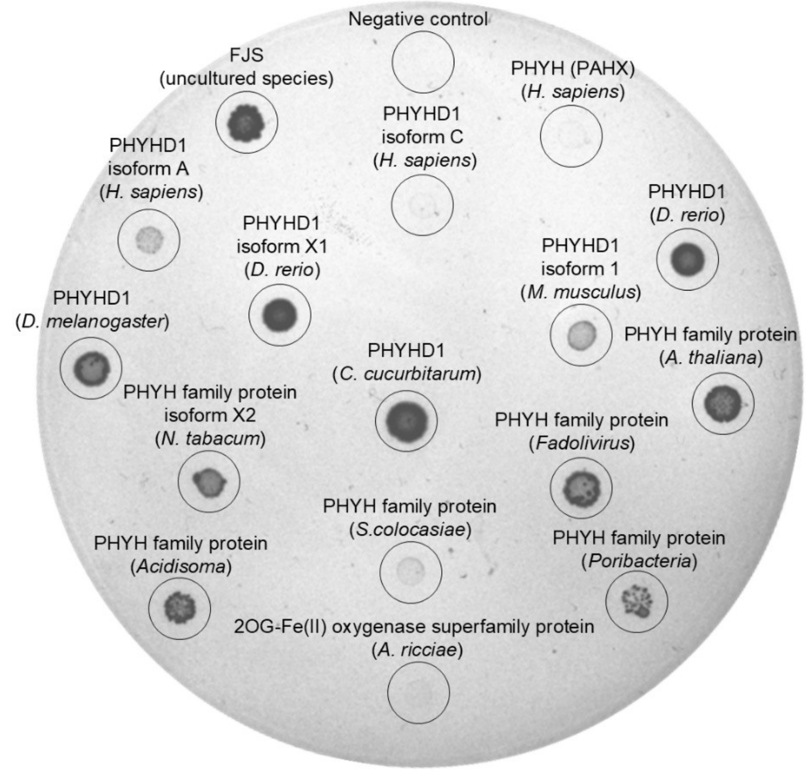

Supplementary Figure S7. Demethylation activity of phytanoil-CoA family oxygenases demonstrated by genetic complementation. Demethylation activity was assessed via complementation of a uridine/cytidine auxotrophic *E. coli* HMS174(DE3) Δ*pyrF* strain after one week of growth at room temperature. Tested oxygenase genes were cloned into either pET-21 or pLATE31 vectors. Abbreviated names of the tested oxygenases are indicated above the corresponding colonies. M9 minimal medium was supplemented with 10 mg L^{-1} 2′-*O*-methylcytidine.

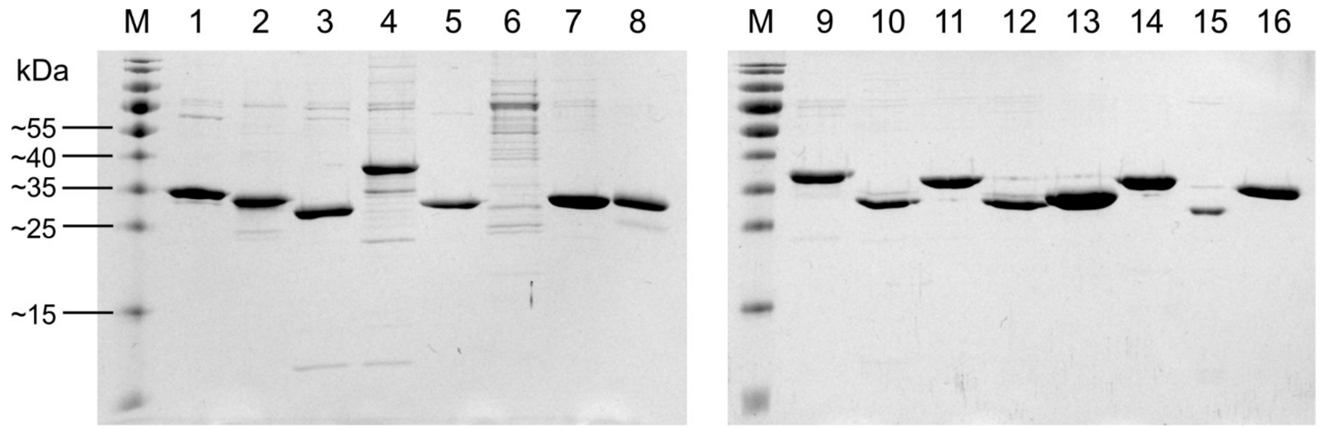

Supplementary Figure S8. SDS-PAGE analysis of different recombinant proteins purified in this study:
M – protein molecular weight marker.
1 – FJS
2 – Phytanoil-CoA dioxygenase family protein (*Acidisoma*)
3 – Phytanoil-CoA dioxygenase family protein (*Poribacteria*)
4 – Phytanoil-CoA dioxygenase (*H. sapiens*)
5 – PHYHD1 isoform A (*H. sapiens*)
6 – PHYHD1 isoform C (*H. sapiens*)
7 – PHYHD1 (*D. rerio*)
8 – PHYHD1 isoform X1 (*D. rerio*)
9 – PHYHD1 isoform 1 (*M. musculus*)
10 – PHYHD1 (*D. melanogaster*)
11 – PHYHD1 (*C. cucurbitarum*)
12 – Phytanoil-CoA dioxygenase (*A. thaliana*)
13 – Phytanoil-CoA dioxygenase family protein isoform X2 (*N. tabacum*)
14 – Phytanoil-CoA dioxygenase family protein (*Fadolivirus algeromassiliense*)
15 – Phytanoil-CoA dioxygenase family protein (*S. colocasiae)*
16 – Unnamed 2OG-Fe(II) oxygenase superfamily protein (*A. ricciae*)

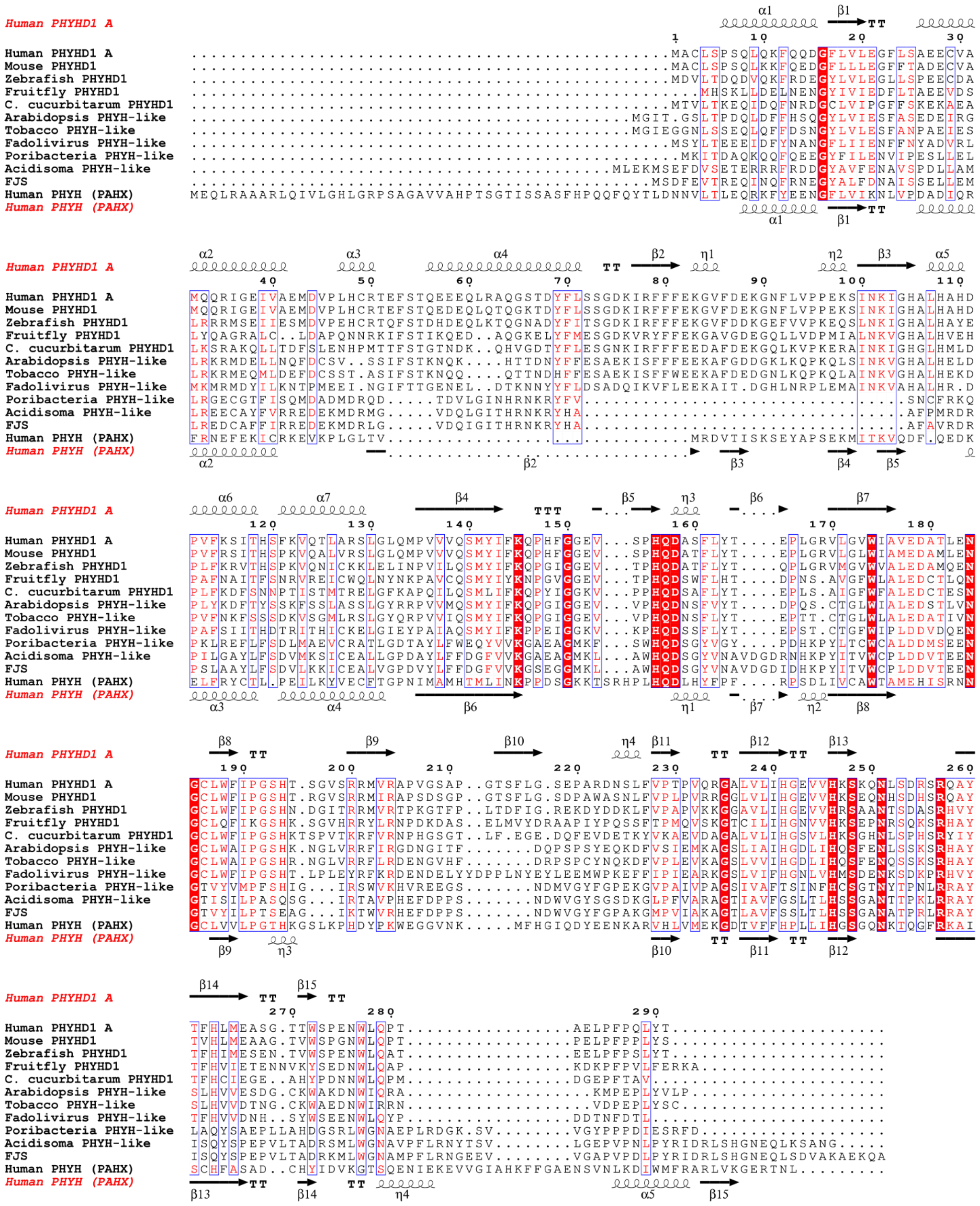

Supplementary Figure S9. Sequence alignment of human PHYH (PAHX) with PHYHD1 and PHYH-like oxygenases found active as 2′-*O*-methyl nucleoside demethylases in vitro. Secondary structure elements from human PHYHD1 isoform A (PDB ID: 3OBZ) (2) are displayed above the alignment, those from human PHYH (PDB ID: 2A1X) (1) are shown below. Multiple sequence alignment was performed using the ClustalW algorithm (3) and visualised with ESPript 3 (4).

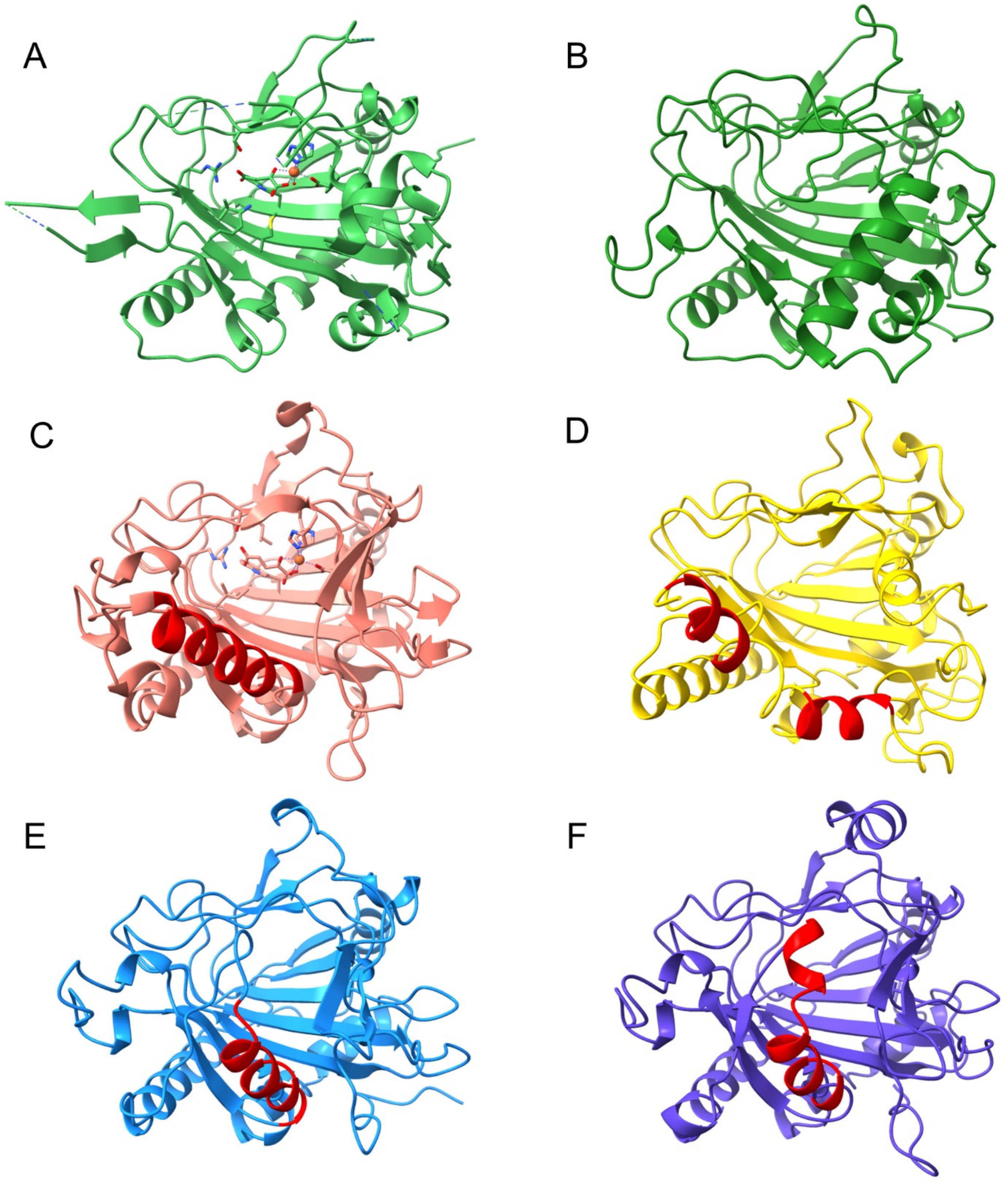

Supplementary Figure S10. Structural comparison of human PHYH (crystal structure, PDB ID: 2A1X (1), **A**), PHYH (predicted structure, **B**), human PHYHD1 (crystal structure, PDB ID: 3OBZ (2), **C**), FJS (predicted structure, **D**), and PHYH-like oxygenases from *Arabidopsis thaliana* (predicted structure, **E**) and *Fadolivirus algeromassiliense* (predicted structure, **F**). Secondary α-helices located near the opening of the active site cavity are highlighted in red. Dotted lines indicate disordered regions present in the PHYH crystal structure. Predicted structures were generated using AlphaFold 3 (5) via the AlphaFold Server.

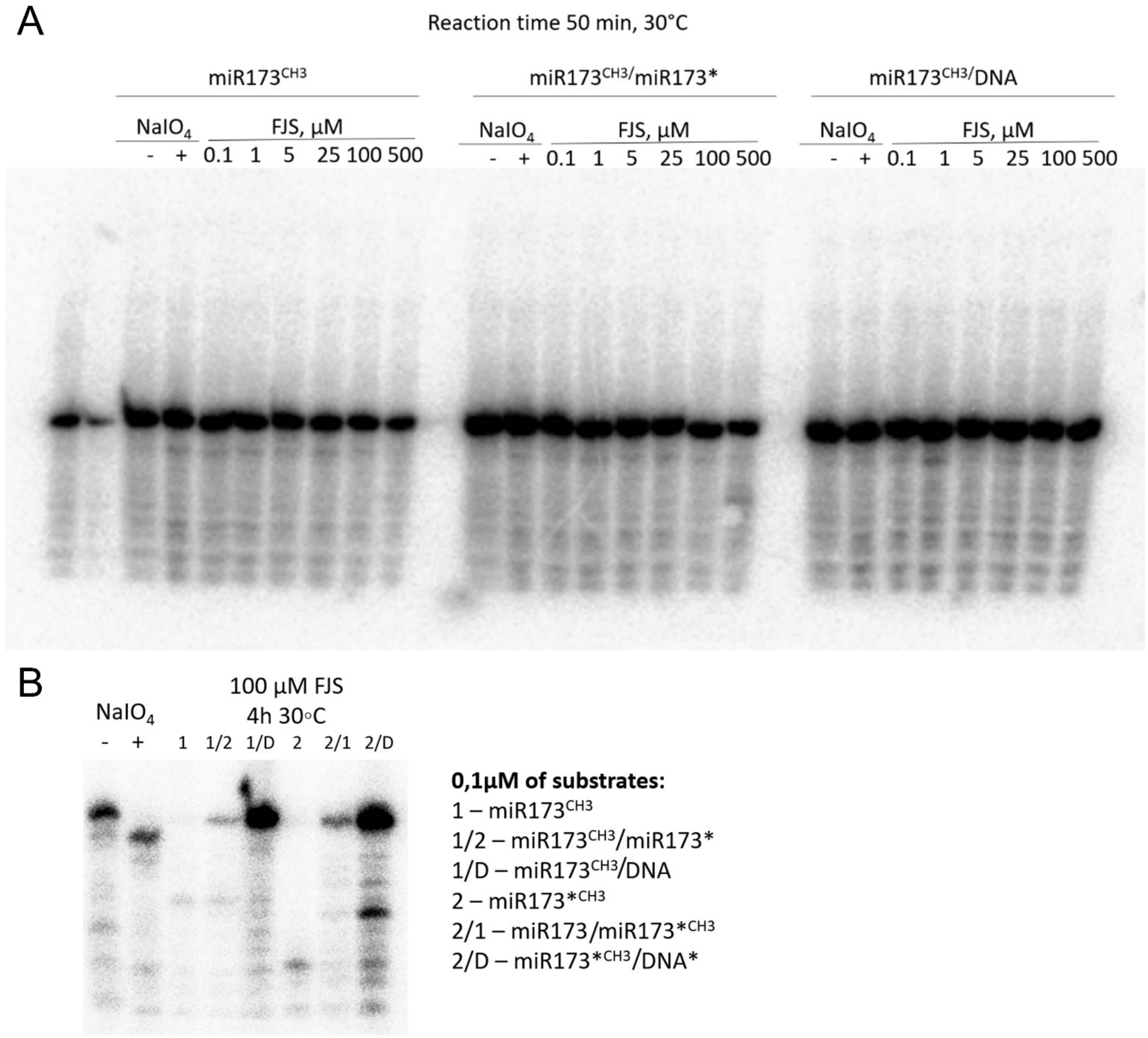

Supplementary Figure S11. (**A**) miRNA 3′-terminal demethylation assay with FJS by sodium periodate ($NaIO_4$)-mediated oxidation followed by β-elimination using single stranded miR173, miR173 duplex with a complementary miR173* strain, and miR173 duplex with a complementary DNA. (**B**) miRNA 3′-terminal demethylation assay with FJS, incubation time increased to 4 hours. Reaction mixtures contained 0.1 µM ssRNA, dsRNA or RNA/DNA duplex, varying amounts of FJS, 10 mM 2-oxoglutarate, 1 mM ascorbate, and 0.25 mM $FeSO_4$ in 50 mM Tris-HCl, pH 8.0.

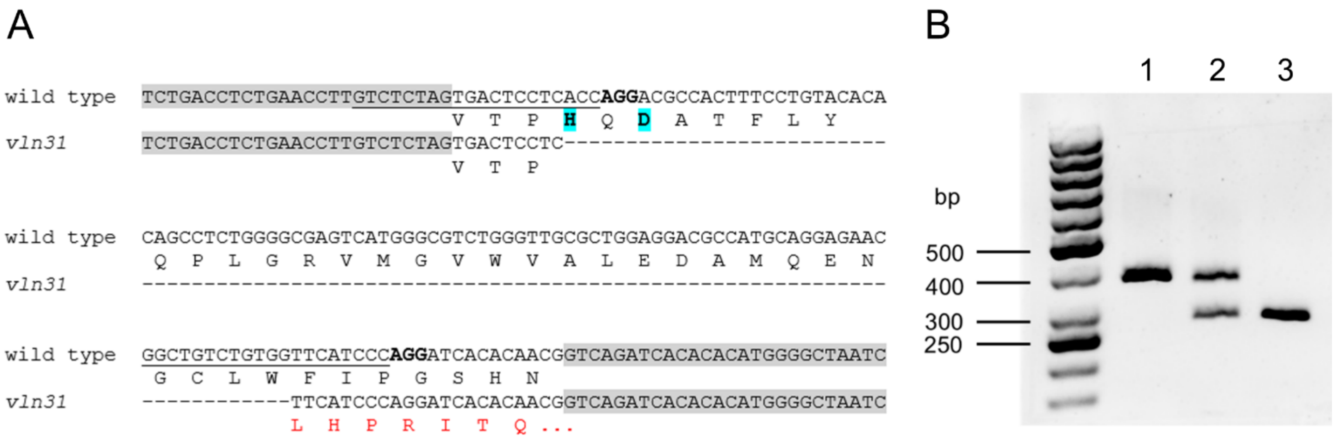

Supplementary Figure S12. Generation and genotyping of the zebrafish *phyhd1* knockout (*vln31*) mutant line. (**A**) Sequence of the *vln31* deletion allele. The DNA sequence of the deletion mutant is aligned with the wild-type sequence. sgRNA targets are underlined with PAMs shown in bold, and introns are highlighted in grey. Protein translation is provided below the DNA sequence, with active site amino acids (H156 and D158) shown in bold with aqua highlight. (**B**) Genotyping of wild type (lane 1), heterozygous mutant (lane 2), and homozygous (lane 3) mutant zebrafish. PCR products were obtained using the phyhd1-D-rerio-genotyping-Fw and phyhd1-D-rerio-genotyping-Rv primers and separated on a 2% agarose gel.

Supplementary Table S2. Sequences of recombinant proteins investigated in this study.

FJS	MSDFEVTREQINQFRNEGYALFDNAISSELLEMLREDCAFFIRREDEKMDRLGVDQIGITHRNKRYHAAFAVRDRPSLAAYLFSDVLKKICEALVG<br>PDVYLFFDGFVVKGSEGGMKLAWHQDSGYVNAVDGDIDHKPYITVWCPLDDVTEENGTVYILPTSEAGIKTWVRHEFDPPSNDWVGYFGPAKGMPV<br>IAKAGTLAVFSSLTLHSSGANATPRLRRAYISQYSPEPVLTADRKMLWGNAMPFLRNGEEVVGAPVPDLPYRIDRLSHGNEQLSDVAKAEKQAGHH<br>HHHHG
Phytanoil-CoA dioxygenase (*H. sapiens*)	MGQRPGGVGVPRAAAMEQLRAAARLQIVLGHLGRPSAGAVVAHPTSGTISSASFHPQQFQYTLDNNVLTLEQRKFYEENGFLVIKNLVPDADIQRF<br>RNEFEKICRKEVKPLGLTVMRDVTISKSEYAPSEKMITKVQDFQEDKELFRYCTLPEILKYVECFTGPNIMAMHTMLINKPPDSGKKTSRHPLHQD<br>LHYFPFRPSDLIVCAWTAMEHISRNNGCLVVLPGTHKGSLKPHDYPKWEGGVNKMFHGIQDYEENKARVHLVMEKGDTVFFHPLLIHGSGQNKTQG<br>FRKAISCHFASADCHYIDVKGTSQENIEKEVVGIAHKFFGAENSVNLKDIWMFRARLVKGERTNLLEHHHHHH
PHYHD1 isoform A (*H. sapiens*)	MACLSPSQLQKFQQDGFLVLEGFLSAEECVAMQQRIGEIVAEMDVPLHCRTEFSTQEEEQLRAQGSTDYFLSSGDKIRFFFEKGVFDEKGNFLVPP<br>EKSINKIGHALHAHDPVFKSITHSFKVQTLARSLGLQMPVVVQSMYIFKQPHFGGEVSPHQDASFLYTEPLGRVLGVWIAVEDATLENGCLWFIPG<br>SHTSGVSRRMVRAPVGSAPGTSFLGSEPARDNSLFVPTPVQRGALVLIHGEVVHKSKQNLSDRSRQAYTFHLMEASGTTWSPENWLQPTAELPFPQ<br>LYTLEHHHHHH
PHYHD1 isoform C (*H. sapiens*)	MACLSPSQLQKFQQDGFLVLEGFLSAEECVAMQQRIGEIVAEMDVPLHCRTEFSTQEEEQLRAQGSTDYFLSSGDKIRFFFEKGVFDEKGNFLVPP<br>EKSINKIGHALHAHDPVFKSITHSFKVQQPHFGGEVSPHQDASFLYTEPLGRVLGVWIAVEDATLENGCLWFIPGSHTSGVSRRMVRAPVGSAPGT<br>SFLGSEPARDNSLFVPTPVQRGALVLIHGEVVHKSKQNLSDRSRQAYTFHLMEASGTTWSPENWLQPTAELPFPQLYTLEHHHHHH
PHYHD1 (*D. rerio*)	MDVLTDQDVQKFRDEGYLVLEGLLSPEECDALRRRMSEIIESMDVPEHCRTQFSTDHDEQLKTQGNADYFITSGDKIRFFFEKGVFDDKGEFVVPK<br>EQSLNKIGHALHAYEPLFKRVTHSPKVQNICKKLELINPVILQSMYIFKQPGIGGEVTPHQDATFLYTQPLGRVMGVWVALEDAMQENGCLWFIPG<br>SHNDGITRRMVRTPKGTFPLTDFIGREKDYDDKLFVPAPVKKGGAVLIHGEVVHRSAANTSDASRHVYTFHIMESENTVWSPENWLQATEELPFPS<br>LYTGHHHHHHG
PHYHD1 isoform X1 (*D. rerio*)	MDVLTDQDVQKFRDEGYLVLEGLLSPEECDALRRRMSEIIESMDVPEHCRTQFSTDHDEQLKTQMQGNADYFITSGDKIRFFFEKGVFDDKGEFVV<br>PKEQSLNKIGHALHAYEPLFKRVTHSPKVQNICKKLELINPVILQSMYIFKQPGIGGEVTPHQDATFLYTQPLGRVMGVWVALEDAMQENGCLWFI<br>PGSHNDGITRRMVRTPKGTFPLTDFIGREKDYDDKLFVPAPVKKGGAVLIHGEVVHRSAANTSDASRHVYTFHIMESENTVWSPENWLQATEELPF<br>PSLYTGHHHHHHG
PHYHD1 isoform 1 (*Mus musculus*)	MKENSPGAIELSQLSPAVFPMACLSPSQLKKFQEDGFLLLEGFFTADECVAMQQRIGEIVAEMDVPLHCRTEFSTQEDEQLQTQGKTDYFLSSGDK<br>IRFFFEKGVFDEKGNFLVPPEKSINKIGHALHAHDPVFRSITHSPKVQALVRSLGLQMPVVVQSMYIFKQPHFGGEVSPHQDATFLYTEPLGRVLG<br>LWIAMEDAMLENGCLWFIPGSHTRGVSRRMIRAPSDSGPGTSFLGSDPAWASNLFVPLPVRRGGLVLIHGEVVHKSEQNHSDHSRQAYTVHLMEAA<br>GTVWSPGNWLQPTPELPFPPLYSLEHHHHHH
PHYHD1 (*D. melanogaster*)	MHSKLLDELNENGYIVIEDFLTAEEVDSLYQAGRALCLDAPQNNRKIFSTIKQEDAQGKELYFMESGDKVRYFFEKGAVGDEGQLLVDPMIALNKV<br>GHALHVEHPAFNAITFSNRVREICWQLNYNKPAVCQSMYIYKNPGVGGEVTPHQDSWFLHTDPNSAVGFWLALEDCTLQNGCLQFIKGSHKSGVHR<br>RYLRNPDKDASELMVYDRAAPIYPQSSFTPMQVSKGTCILIHGNVVHKSEPNRSQKSRHAYTFHVIETENNVKYSEDNWLQAPKDKPFPVLFERKA<br>LEHHHHHH
PHYHD1 (*C.cucurbitarum*)	MTVLTKEQIDQFNRDGCLVIPGFFSKEKAEALKSRAKQLLTDFSLENHPMTTFSTGTNDKQHVGDTYFLESGNKIRFFFEEDAFDEKGQLKVPKER<br>AINKIGHGLHMLDPLFKDFSNNPTISTMTRELGFKAPQILQSMLIFKQPYIGGKVPPHQDSAFLYTEPLSAIGFWFALEDCTESNGCLWFIPGSHK<br>TSPVTKRFVRNPHGSGTLFEGEDQFEVDETKYVKAEVDAGALVLIHGSVLHKSGHNLSPHSRYIYTFHCIEGEAHYPDNNWLQPMDGEPFTAVLEH<br>HHHHH
PHYH family protein (*A.thaliana*)	MGITGSLTPDQLDFFHSQGYLVIESFASEDEIRGLRKRMDELLNQFDCSVSSIFSTKNQKHTTDNYFFESAEKISFFFEEKAFGDDGKLKQPKQLS<br>INKVGHALHELDPLYKDFTYSSKFSSLASSLGYRRPVVMQSMYIFKQPGIGGEVVPHQDNSFVYTDPQSCTGLWIALEDSTLVNGCLWAIPGSHKN<br>GLVRRFIRGDNGITFDQPSPSYEQKDFVSIEMKAGSLIAIHGDLIHQSFENLSSKSRHAYSLHVVESDGCKWAKDNWIQRAKMPEPLYVLPLEHHH<br>HHH
PHYH family protein isoform X2 (*N. tabacum*)	MGIEGGNLSSEQLQFFDSNGYLVLESFANPAEIESLRKRMEQMLDEFDCSSTASIFSTKNQQQTTNDHFFESAEKISFFWEEKAFDEDGNLKQPKQ<br>LAINKVGHALHEKDPVFNKFSSSDKVSGMLRSLGYQRPVVIQSMYIFKQPGIGGEVVPHQDNSFLYTEPTTCTGLWLALEDATIVNGCLWAIPGSH<br>RNGLVRRFLRDENGVHFDRPSPCYNQKDFVPLEVKAGSLVVIHGDLIHQSFENQSSKSRHAYSLHVVDTNGCKWAEDNWIRRNVDPEPLYSCLEHH<br>HHHH
PHYH family protein (*Fadolivirus algeromassiliense*)	MSYLTEEEIDFYNANGFLIIENFFNYADVRLMKMRMDYILKNTPMEEINGIFTTGENELDTKNNYYFLDSADQIKVFLEEKAITDGHLNRPLEMAI<br>NKVAHALHRDPAFSIITHDTRITHICKELGIEYPAIAQSMYIFKPPEIGGKVKPHQDSSFLYTEPSTCTGFWIPLDDVDQENGCLWFIPGSHTLPL<br>EYRFKRDENDELYYDPPLNYEYLEEMWPKEFFIPIEARKGSLVIFHGNLVHMSDENKSDKPRHVYTFHVVDNHSYWSEENWLQYPDDTNFDTLLEH<br>HHHHH
PHYH family protein (*Acidisoma*)	MLEKMSEFDVSETERRRFRDDGYAVFENAVSPDLLAMLREECAYFVRREDEKMDRMGVDQLGITHRNKRYHAAFPMRDRPILGAYLFSDVMKSICE<br>ALLGPDAYLFFDGFVVKGAEAGMKLAWHQDSGYVNAVDGDRNHKPYITVWCPLDDVTEENGTISILPASQSGIRTAVPHEFDPPSNDWVGYSGSDK<br>GLPFVARAGTIAVFGSLTLHSSGANTTPKLRRAYISQYSPEPVLTADRSMLWGNAVPFLRNYTSVLGEPVPNLPYRIDRLSHGNEQLKSANGLEHH<br>HHHH
PHYH family protein (*S. colocasiae*)	MDYSAQRAQFAAEGYAVFERVLEGPLLDLLRDECGRVIEREDARLDALGVEVDGISHKGKRYFAGECQRVQPELRVMLFSETMADICRATLGDDAY<br>FFYDQYVVKGADKGMPFSWHQDSGYVVGNGGPPDHKPYLTCWCTLDDTTVANGTVRILPFSQVPATRDGIVPHERQPGSNDLVGYSGDAEGVTLEV<br>PAGSVVAFSSLALHATGSNTTPKMRRVYLAQYSPEPILNPGTNHLRRNAIAFLRGGRQVTISLEHHHHHH
PHYH family protein (*Poribacteria*)	MKITDAQKQQFQEEGYFILENVIPESLLELLRGECGTFISQMDADMDRQDTDVLGINHRNKRYFVSNCFRKQPKLREFLFSDLMAEVCRATLGDTA<br>YLFWEQYVVKGAEAGMKFSWHQDSGYVGYPDHKPYLTCWCALDDMSEENGTVYVMPFSHIGIRSWVKHVREEGSNDMVGYFGPEKGVPAIVPAGSI<br>VAFTSINFHCSGTNYTPNLRRAYLAQYSAEPLLAHDGSRLWGNAEPLLRDGKSVVGYPPPDIESRFDLEHHHHHH
Unnamed 2OG-Fe(II) oxygenase superfamily protein (*A. ricciae*)	MSNMNSMLVTSEHLKQFDEHGYFILPSIISQEHLHMMNEIIQRAIAKRDEDLKNEVNKDGLIQCSTDGKYFFFMHEDHEPEMKKIIFSSYMEQITK<br>AILGEKVYYTHSEIVVKRGEGEKRDFGERTKFSWHQDSGYVPYKHTPYLSCWCALTEMTSTNGTISVLPVERNPPEDKHHHINWSGKPRPSAHIEF<br>PCYQHRKDESSPDLIGYFGTDRGVEVTCPAGSIVVFSSLTLHCSSANRSNALRSAYNIQYAPVPLMSEDNQSFRHKADPFIVQSETIDETNQLEHH<br>HHHH

Supplementary Table S3. Genome-wide association studies linking genetic variation in PHYHD1 to the levels of 2′-O-methylcytidine and 2′-O-methyluridine. Data were obtained from the NHGRI-EBI GWAS Catalog (6) The table lists studies reporting associations between PHYHD1 variants and altered levels of Cm and Um: R – not reported, RAF – risk allele frequency, Cm – 2′-*O*-methylcytidine, Um – 2′-*O*-methyluridine.

INITIAL SAMPLE SIZE	SAMPLE	METABOLITE	REGION	POSITION	MAPPED GENE(S)	STRONGEST SNP-RISK ALLELE	CONTEXT	RAF	P-VALUE	BETA VALUE [95% CI]	INCREASE OR DECREASE	REF.
1,221 cases of chronic kidney disease	Urine	Cm	9q34.11	128922736	PHYHD1	rs55758160-G	intron variant	0.39	1.00E-55	0.302 [0.26-0.34]	unit increase	(7)
		Um	9q34.11	128937261	PHYHD1	rs12555901-T	intron variant	0.39	3.00E-37	0.626 [0.53-0.72]	unit increase	
3,926 Hispanic/Latino individuals	Serum	Cm	9q34.11	128922557	PHYHD1	rs57294583-A	intron variant	0.548395	4.00E-189	0.668 [0.62-0.71]	unit decrease	(8)
	Serum	Um	9q34.11	128922557	PHYHD1	rs57294583-A	intron variant	0.548395	2.00E-230	0.738 [0.69-0.78]	unit decrease	
	Serum	Um	9q34.11	128938669	PHYHD1	rs17507852-C	intron variant	0.945615	2.00E-38	0.692 [0.59-0.8]	unit decrease	
4,947 European ancestry individuals	Plasma	Cm	9q34.11	128922736	PHYHD1	rs55758160-G	intron variant	0.4	3.00E-155	0.228 [0.21-0.25]	unit increase	(9)
4,899 European ancestry individuals	Urine	Cm	9q34.11	128922736	PHYHD1	rs55758160-G	intron variant	0.4	1.00E-120	0.293 [0.27-0.32]	unit increase	
3,855 European ancestry individuals	Urine	Um	9q34.11	128924345	PHYHD1	rs55868600-A	intron variant	0.43	3.00E-100	0.429 [0.39-0.47]	unit increase	
619 African American individuals	Serum	Um	9q34.11	128922736	PHYHD1	rs55758160 - ?	intron variant	NR	2.00E-42	0.72 [NR]	unit increase	(10)
	Serum	Cm	9q34.11	128922557	PHYHD1	rs57294583 - ?	intron variant	NR	2.00E-12	0.39 [NR]	unit increase	
291 European ancestry individuals	Cerebrospinal fluid	Cm	9q34.11	128922557	PHYHD1	rs57294583-A	intron variant	0.5961	7.00E-44	0.121 [NR]	unit decrease	(11)
1,170 Israeli ancestry individuals	Serum	Um	9q34.11	128940612	PHYHD1	rs2273866-?	missense variant	NR	5.00E-22	1.08 [0.87-1.29]	unit increase	(12)
	Serum	Cm	9q34.11	128940612	PHYHD1	rs2273866-?	missense variant	NR	1.00E-17	0.662 [0.52-0.81]	unit increase	
2,466 Black/admixed ancestry individuals	Plasma	Cm	9q34.11	128922557	PHYHD1	rs57294583-G	intron variant	NR	7.00E-117	0.691 [0.63-0.75]	unit increase	(13)
6,160 European, Hispanic or African American individuals	Plasma	Cm	9q34.11	128922361	PHYHD1	rs73624815-A	splice donor 5th base variant	0.0158	6.00E-25	0.764 [0.62-0.91]	unit increase	(14)
	Plasma	Cm	9q34.11	128922557	PHYHD1	rs57294583-G	intron variant	0.4317	1.00E-294	0.674 [0.64-0.71]	unit increase	
5,797 European, Hispanic or African American individuals	Plasma	Um	9q34.11	128922557	PHYHD1	rs57294583-G	intron variant	0.4437	5.00E-303	0.732 [0.7-0.77]	unit increase	(14)
7,582 European ancestry individuals	Plasma	Um	9q34.11	128930572	PHYHD1	rs28373373-C	intron variant	0.396283	2E-504	0.688 [0.66-0.72]	unit increase	(15)
8,122 European ancestry individuals	Plasma	Cm	9q34.11	128942154	PHYHD1 - DOLK	rs10988162-C	intron variant	0.389115	3E-433	0.625 [0.6-0.65]	unit increase	
2,602 European ancestry individuals	Cerebrospinal fluid	Cm	9q34.11	128944776	PHYHD1 - DOLK	rs9299324-T	intron variant	NR	8.00E-171	0.117 [0.11-0.13]	unit decrease	(16)
490 Athletic individuals	Serum	Um	9q34.11	128943484	PHYHD1 - DOLK	rs17432839-?	intron variant	NR	3.00E-10	0.397 [0.28-0.52]	unit increase	(17)

SUPPLEMENTARY REFERENCES

1. McDonough,M.A., Kavanagh,K.L., Butler,D., Searls,T., Oppermann,U. and Schofield,C.J. (2005) Structure of Human Phytanoyl-CoA 2-Hydroxylase Identifies Molecular Mechanisms of Refsum Disease. *Journal of Biological Chemistry*, **280**, 41101–41110.

2. Zhang,Z., Kochan,G.T., Ng,S.S., Kavanagh,K.L., Oppermann,U., Schofield,C.J. and McDonough,M.A. (2011) Crystal structure of PHYHD1A, a 2OG oxygenase related to phytanoyl-CoA hydroxylase. *Biochemical and Biophysical Research Communications*, **408**, 553–558.

3. Thompson,J.D., Higgins,D.G. and Gibson,T.J. (1994) CLUSTAL W: improving the sensitivity of progressive multiple sequence alignment through sequence weighting, position-specific gap penalties and weight matrix choice. *Nucleic Acids Res*, **22**, 4673–4680.

4. Robert,X. and Gouet,P. (2014) Deciphering key features in protein structures with the new ENDscript server. *Nucleic Acids Res*, **42**, W320–W324.

5. Abramson,J., Adler,J., Dunger,J., Evans,R., Green,T., Pritzel,A., Ronneberger,O., Willmore,L., Ballard,A.J., Bambrick,J., et al. (2024) Accurate structure prediction of biomolecular interactions with AlphaFold 3. *Nature*, **630**, 493–500.

6. Cerezo,M., Sollis,E., Ji,Y., Lewis,E., Abid,A., Bircan,K.O., Hall,P., Hayhurst,J., John,S., Mosaku,A., et al. (2025) The NHGRI-EBI GWAS Catalog: standards for reusability, sustainability and diversity. *Nucleic Acids Research*, **53**, D998–D1005.

7. Schlosser,P., Li,Y., Sekula,P., Raffler,J., Grundner-Culemann,F., Pietzner,M., Cheng,Y., Wuttke,M., Steinbrenner,I., Schultheiss,U.T., et al. (2020) Genetic studies of urinary metabolites illuminate mechanisms of detoxification and excretion in humans. *Nat Genet*, **52**, 167–176.

8. Feofanova,E.V., Chen,H., Dai,Y., Jia,P., Grove,M.L., Morrison,A.C., Qi,Q., Daviglus,M., Cai,J., North,K.E., et al. (2020) A Genome-wide Association Study Discovers 46 Loci of the Human Metabolome in the Hispanic Community Health Study/Study of Latinos. *Am J Hum Genet*, **107**, 849–863.

9. Schlosser,P., Scherer,N., Grundner-Culemann,F., Monteiro-Martins,S., Haug,S., Steinbrenner,I., Uluvar,B., Wuttke,M., Cheng,Y., Ekici,A.B., et al. (2023) Genetic studies of paired metabolomes reveal enzymatic and transport processes at the interface of plasma and urine. *Nat Genet*, **55**, 995–1008.

10. Luo,S., Feofanova,E.V., Tin,A., Tung,S., Rhee,E.P., Coresh,J., Arking,D.E., Surapaneni,A., Schlosser,P., Li,Y., et al. (2021) Genome-wide association study of serum metabolites in the African American Study of Kidney Disease and Hypertension. *Kidney Int*, **100**, 430–439.

11. Panyard,D.J., Kim,K.M., Darst,B.F., Deming,Y.K., Zhong,X., Wu,Y., Kang,H., Carlsson,C.M., Johnson,S.C., Asthana,S., et al. (2021) Cerebrospinal fluid metabolomics identifies 19 brain-related phenotype associations. *Commun Biol*, **4**, 63.

12. Bar,N., Korem,T., Weissbrod,O., Zeevi,D., Rothschild,D., Leviatan,S., Kosower,N., Lotan-Pompan,M., Weinberger,A., Le Roy,C.I., et al. (2020) A reference map of potential determinants for the human serum metabolome. *Nature*, **588**, 135–140.

13. Tahir,U.A., Katz,D.H., Avila-Pachecho,J., Bick,A.G., Pampana,A., Robbins,J.M., Yu,Z., Chen,Z.-Z., Benson,M.D., Cruz,D.E., et al. (2022) Whole Genome Association Study of the Plasma Metabolome Identifies Metabolites Linked to Cardiometabolic Disease in Black Individuals. *Nat Commun*, **13**, 4923.

14. Feofanova,E.V., Brown,M.R., Alkis,T., Manuel,A.M., Li,X., Tahir,U.A., Li,Z., Mendez,K.M., Kelly,R.S., Qi,Q., et al. (2023) Whole-Genome Sequencing Analysis of Human Metabolome in Multi-Ethnic Populations. *Nat Commun*, **14**, 3111.

15. Chen,Y., Lu,T., Pettersson-Kymmer,U., Stewart,I.D., Butler-Laporte,G., Nakanishi,T., Cerani,A., Liang,K.Y.H., Yoshiji,S., Willett,J.D.S., et al. (2023) Genomic atlas of the plasma metabolome prioritizes metabolites implicated in human diseases. *Nat Genet*, **55**, 44–53.

16. Wang,C., Yang,C., Western,D., Ali,M., Wang,Y., Phuah,C.-L., Budde,J., Wang,L., Gorijala,P., Timsina,J., et al. (2024) Genetic architecture of cerebrospinal fluid and brain metabolite levels and the genetic colocalization of metabolites with human traits. *Nat Genet*, **56**, 2685–2695.

17. Al-Khelaifi,F., Diboun,I., Donati,F., Botrè,F., Abraham,D., Hingorani,A., Albagha,O., Georgakopoulos,C., Suhre,K., Yousri,N.A., et al. (2019) Metabolic GWAS of elite athletes reveals novel genetically-influenced metabolites associated with athletic performance. s*Sci Rep*, **9**, 19889.
